# Exploring People’s Candidacy for Mobile Health–Supported HIV Testing and Care Services in Rural KwaZulu-Natal, South Africa: Qualitative Study

**DOI:** 10.2196/15681

**Published:** 2019-11-18

**Authors:** Oluwafemi Adeagbo, Carina Herbst, Ann Blandford, Rachel McKendry, Claudia Estcourt, Janet Seeley, Maryam Shahmanesh

**Affiliations:** 1 Africa Health Research Institute KwaZulu-Natal Mtubatuba South Africa; 2 University College London London United Kingdom; 3 University of Johannesburg Auckland Park Johannesburg South Africa; 4 Glasgow Caledonian University London United Kingdom; 5 London School of Hygiene and Tropical Medicine London United Kingdom

**Keywords:** mHealth, antiretroviral therapy, HIV testing, South Africa, candidacy framework

## Abstract

**Background:**

The use of mobile communication technologies (mHealth: mobile health) in chronic disease management has grown significantly over the years. mHealth interventions have the potential to decentralize access to health care and make it convenient, particularly in resource-constrained settings. It is against this backdrop that we aimed to codevelop (with potential users) a new generation of mobile phone–connected HIV diagnostic tests and Web-based clinical care pathways needed for optimal delivery of decentralized HIV testing, prevention, and care in low- and middle-income countries.

**Objective:**

The aim of this study was to understand ways in which an mHealth intervention could be developed to overcome barriers to existing HIV testing and care services and promote HIV self-testing and linkage to prevention and care in a poor, HIV hyperendemic community in rural KwaZulu-Natal, South Africa.

**Methods:**

A total of 54 in-depth interviews and 9 focus group discussions were conducted with potential users (including health care providers) in 2 different communities. Theoretically informed by the candidacy framework, themes were identified from the interview transcripts, manually coded, and thematically analyzed.

**Results:**

Participants reported barriers, such as fear of HIV identity, stigma, long waiting hours, clinic space, and health care workers’ attitudes, as major impediments to effective uptake of HIV testing and care services. People continued to reassess their candidacy for HIV testing and care services on the basis of their experiences and how they or others were treated within the health systems. Despite the few concerns raised about new technology, mobile phone–linked HIV testing was broadly acceptable to potential users (particularly men and young people) and providers because of its privacy (individual control of HIV testing over health provider–initiated testing), convenience (individual time and place of choice for HIV testing versus clinic-based testing), and time saving.

**Conclusions:**

Mobile phone–connected HIV testing and Web-based clinical care and prevention pathways have the potential to support access to HIV prevention and care, particularly for young people and men. Although mHealth provides a way for individuals to test their candidacy for HIV services, the barriers that can make the service unattractive at the clinic level will also need to be addressed if potential demand is to turn into actual demand.

## Introduction

### HIV Burden in South Africa and KwaZulu-Natal Province

The United Nations’ Sustainable Development Goals herald a major commitment to accelerate the pace of progress made in tackling the HIV epidemic [1]. However, despite huge advances in antiretroviral therapies (ARTs) and antiretroviral-based prevention, many countries are struggling to reach this target, as they do not have the tools or mechanisms to deliver ART at scale to people in need [2,3]. Globally, South Africa (SA) has the highest number of people living with HIV (People living with HIV: estimated 7.9 million), with 20% of the total global antiretrovirals use and an HIV prevalence rate of 14% in 2017 [4,5]. With its large HIV prevention programs, SA has made significant progress in reducing AIDS-related mortality and the number of new HIV infections. However, annual new HIV infections (231,000 in 2017) are still high [4].

The province of KwaZulu-Natal (KZN) is disproportionately affected by the epidemic, with an estimated 18.1% prevalence rate [4], whereas in the uMkhanyakude district in the north of the province, the site of the research reported in this paper, it is estimated at 30% in the general population, with 5% to 7% annual HIV incidence rate in young women and men [6]. Furthermore, there is a high HIV-related mortality rate in men and less than 50% annual HIV testing uptake among men under 29 years, and only 50% of those diagnosed in the general population reach clinical services within 1 year. At the same time, clinic attendances for treatment have risen by 300% over the past 6 years, placing huge pressures on health systems, with escalating costs [6,7]. Therefore, there is a need to develop interventions that can close the gap in HIV testing, prevention, and treatment, to reduce HIV incidence and mortality, while reducing the current pressures on primary health care clinics. In this study, we sought to understand the current barriers and facilitators to HIV testing and care services to codevelop with potential users a mobile health (mHealth) intervention that will aid people’s engagement with HIV care services. We used the *candidacy framework*, described below, to structure our findings. Moreover, the proposed mHealth intervention is described below.

### The Limit of Universal HIV Test and Treat and HIV Prevention Technologies

Universal HIV test and treat (UTT) and newer HIV prevention technologies, such as Pre-Exposure Prophylaxis (PrEP), have the potential to be game changers, but all require people to know their HIV status and engage in the cascade of care and prevention [7-10]. A treatment as prevention (TasP) trial conducted in rural SA failed to show an effect, partly because of social and structural barriers to link men and young people to care [7,11]. Similarly, a *test and start* study in Eswatini found that men still delay HIV testing and treatment until they have advanced disease; this is because of social barriers and stigma associated with accessing HIV care [12]. Although there was an effect on HIV incidence, the HPTN 071 trial (POPART) shows limited linkage to care for young men and women [13]. Despite the potential of UTT to halt HIV transmission, balancing social realities and science is very important to understand the context of HIV prevention across different settings [12,14]. Although stigma and other factors have been a major barrier to HIV care [12], research has shown that various HIV prevention technologies, such as HIV self-testing (HIVST), have the potential to widen access to testing, especially for hard-to-reach groups, such as adolescents and men [15-17].

However, for the game-changing potential of new innovations to be achieved in high-burden settings, such as SA, there is a need for those who test positive to link into immediate ART, as well as those who test negative to access combinations of HIV prevention interventions tailored to keep them negative, including but not limited to condoms, PrEP, and voluntary male circumcision. Therefore, there is an urgent need to identify cost-effective and acceptable technologies for population-based HIV testing, including self-testing with linkage and retention in care.

### The Potential of Mobile Health in Closing the Gaps in the HIV Treatment Cascade

**Figure 1:**
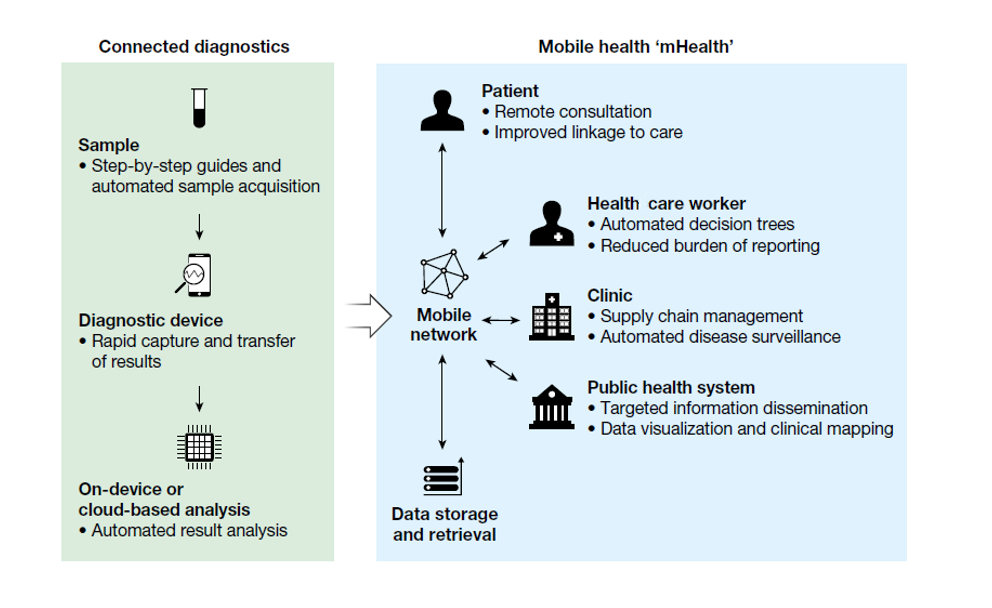
Mobile health–connected diagnostics by Wood et al, 2019.

The use of mobile communication technologies (mHealth) in chronic disease management has grown significantly over the years. mHealth interventions have the potential to decentralize access to health care and make it convenient, particularly in resource-constrained settings [18-22]. It is well documented that mHealth interventions, such as SMS text messaging, have been used to support management of diseases, such as hypertension and diabetes, and to support HIV treatment [20,23,24]. In fact, the World Health Organization recommended the use of mobile phone–technologies for ART adherence and other chronic disease management [25]. With 76 million mobile phone subscribers, SA has the highest penetration rate in Africa, therefore it has potential for mHealth interventions [26,27]. Current innovations in HIVST, task shifting to community caregivers, and the rise in HIV treatment adherence clubs, try to support chronic disease management and reduce the pressure on health facilities by shifting the focus of HIV testing, treatment, and prevention away from health care facilities to the community. However, in the absence of robust and safe clinical pathways to support these interventions, their reach will be limited. There is now an opportunity with the growth in mobile technologies to provide digital support for these innovations (see Figure 1), while pushing the boundaries further to potentially supporting the entire continuum of HIV care, from primary prevention to long-term treatment and improved sexual health of people living with HIV.

SA has a progressive mHealth strategy [27] to deliver improved HIV care services, but this emerging field is still in its infancy, with currently no safe and acceptable Web-based clinical care pathways and very limited knowledge of the feasibility and acceptability of implementing these technologies within existing care pathways. mHealth interventions are complex interventions that need to involve prospective users and service providers (SPs) in their development—considering the patterns and preferences for technology—to maximize uptake and scalability of interventions using mobile electronic devices, such as mobile phones, for the delivery of health care services and avoid inadvertently worsening health inequity [19,23,28]. The mAfrica study aimed to harness this growth in mobile phone usage and potential for enhanced diagnostic tests using inbuilt phone sensors (eg, camera) to interpret and display results and then direct people to local clinics to support virtual follow-up appointments and potentially provide access to antiretrovirals for both treatment and prevention in the community. In summary, this study aimed to codevelop (with potential users) a new generation of mobile phone–connected HIV diagnostic tests and Web-based clinical care pathways needed for optimal delivery of decentralized HIV testing, prevention, and care in low- and middle-income countries. This paper presents the qualitative data from the formative phase of the study to gain insight into potential users’ perceptions of barriers to HIV testing and treatment, as well as their willingness to use the proposed app to promote HIVST and further linkage to care through Web-based clinical care pathways in rural KZN.

## Methods

### Overview

The research was conducted with 2 different communities in a rural subdistrict (228,000 population) of uMkhanyakude district in KZN. The 2 communities were different in terms of their development, access to basic amenities, and housing structures. The first community (CA) is a township that is more developed, with more amenities, population, and modern housing structures, compared with the second community (CB) that is rural in nature, with few basic amenities and housing structures. Each community has government-funded health clinics. The research was conducted by a group of social scientists (males and females) trained in qualitative data collection methods and competent in both IsiZulu (local language) and English. A total of 4 social scientists conducted the community in-depth interviews (IDIs) and focus group discussions (FGDs) in the local language, and a senior social scientist conducted the SPs’ interviews in English. Participants were purposively sampled and recruited individually within the research communities to represent different age groups, gender, and geographical locations in the same district. In our experience, group discussions yield richer data around the normative thinking within demographic groups because of intergenerational and gender relationships if we divide groups by gender and age. This was particularly important, given our aims to explore the ways that different groups may respond differently to the intervention. Thus, community members were divided into 4 categories for IDIs and FGDs: young female, young male, older female, and older male. A total of 4 health care providers (2 female pharmacists and 2 male community HIV testing services providers) participated in IDIs, and 9 research nurses (3 males and 6 females) participated in an FGD together; they were all regarded as SPs. Moreover, both community members and SPs (health care professionals) were regarded as *end users* in this study.

A total of 54 IDIs and 9 FGDs were conducted, with participants (both sexes aged 18-79 years) in both communities, between November 2017 and March 2018. Although our target was 5 IDIs for each group of community members, we slightly exceeded the numbers with some groups and stopped at saturation. The distribution across communities and categories is summarized in Table 1.

**Table 1:** Participant demographics and data collection method.

Population^a,b^		Age range (years) (IDIs^c^)	IDIs conducted (n)	FGDs^d^ conducted (n)	Age range (years) (FGDs)	FGD participants (n)
**Community A (township)**						
	Young female	23-32	6	1	19-32	6
	Young male	18-32	8	1	18-28	7
	Older male	37-46	7	1	35-67	11
	Older female	43-50	6	1	43-67	6
**Community B (rural area)**						
	Young female	18-34	6	1	18-23	10
	Young male	20-33	6	1	19-31	8
	Older male	36-79	6	1	36-58	6
	Older female	35-60	5	1	36-48	6
**Community service providers (SPs) IDIs**						
	Pharmacist (F^e^)	28 (SP1)	1	—^f^	—	—
	Pharmacist (F)	34 (SP2)	1	—	—	—
	HTS^g^/counsellor supervisor (M^h^)	53 (SP3)	1	—	—	—
	HTS counsellor (M)	30 (SP4)	1	—	—	—
**Community service providers FGDs**						
	Research nurses: (F and M)	—	—	1	33-55	9 (6 F and 3 M)

^a^Data collection period: November 2017 to March 2018.

^b^General population: 18 to 79 years.

^c^IDI: in-depth interview.

^d^FGD: focus group discussion.

^e^F: female.

^f^Not applicable.

^g^HTS: HIV testing services

^h^M: male.

All IDIs and FGDs were conducted in participants’ language and places of choice in the community after voluntary informed consent (written and verbal) was obtained. IDIs lasted between 30 and 60 min, whereas FGDs lasted between 90 and 120 min. Through semistructured interviews, we explored participants’ views around barriers to HIV testing and treatment in their communities, as well as their willingness (including their fears and expectations) to use a digital technology to promote HIVST and further linkage to care. Moreover, the group discussions afforded us the opportunity to tease out the complexities, similarities, and differences in what participants said during individual interviews. Moreover, reflective summaries of all interviews conducted, as well as findings, were written down and discussed during several debriefing sessions with other team members. All interviews were digitally recorded, and those conducted in IsiZulu were transcribed verbatim and translated to English for thematic analysis. The data were stored and managed in a Web-based shared drive with limited access. All data were deidentified to protect confidentiality. The data were scrutinized and compared with the recordings by a senior social scientist (OA) and 3 senior research assistant supervisors for quality control. In addition, a 2-day *coding and analysis* workshop was held with the social scientists who conducted the fieldwork. During the workshop, emerging themes were identified, and coding was crosschecked for consistency across coders. Following a *candidacy framework* [29,30], as described below, themes were identified from the interview transcripts, manually coded, and thematically analyzed to present the subjective views of the participants. Key themes discussed in this study were agreed upon by the research team. The study was approved by the Biomedical Research Ethics Committee of the University of KwaZulu-Natal, South Africa (Reference Number: BE435/17).

### Theoretical Framework

The analysis for this paper is based on an adapted *candidacy framework* to explore community barriers and facilitators to HIV testing and treatment, as well as participants’ willingness to use an mHealth technology [29-31]. The *candidacy framework* was originally constructed to investigate the process of accessing health care by vulnerable people in the United Kingdom [29], and it has been modified in different ways over the years, including to examine the utilization of health services by young people in KZN [32]. Taking a candidacy approach places the focus on the choices individuals make, as summarized in Table 2.

Individuals need to first recognize that they are eligible for a health care service and that people like them can access the service. Individuals presenting themselves at health care facilities affirm their candidacy for that service, but their eligibility is adjudicated by health care providers who decide their suitability. Once access has been established, continuing to use health care services requires navigating relevant systems (eg, time off work and transportation) and continuing to consider that the service meets their needs. The pathway to accessing care requires an active response from users and the ability to overcome the barriers [30,32,33]. We use the *candidacy framework* in our data analysis to show the complexities and intersections of multiple factors impeding and supporting individual access to HIV care services in a resource-constrained setting, as well as the ways mHealth-supported HIV testing can or cannot support users to explore their *candidacy* in relation to HIV prevention and care.

**Table 2:** Characteristics of the 7 stages of the candidacy framework by Mackenzie et al.

Stages of candidacy	Description of stages
Identification of candidacy	The process by which individuals come to view themselves as legitimate candidates for particular services
Navigation of services	Knowing how to interact with appropriate services in relation to identified candidacy
Permeability of services	Includes the level of explicit or implicit gatekeeping within a service and the complexity of its referral systems, referring to the *cultural alignment* between users and services
Appearing at services and asserting candidacy	The actions that individuals must take to assert their candidacy in an interaction with a health care professional
Adjudication by professionals	Candidacy, as expressed by service users, is validated or otherwise by health care professionals, which influences subsequent service offers
Offers of, resistance to, services	Emphasizes that follow-up services may be appropriately or inappropriately offered and that these may or may not be acted upon by service users
Operating conditions and local production of candidacy	This incorporates factors that influence decisions about subsequent service provision (eg, the resources available for addressing candidacy) and the kinds of contingent relationships that develop between professionals and service users over a few encounters

## Results

The section is divided into 2 main parts: (1) barriers to the identification of candidacy for HIV testing and care and (2) assessing candidacy through HIVST and mobile phone–enabled linkage to care. The themes under these broad headings engage with key challenges faced by people in identifying their candidacy for HIV testing, treatment or prevention, and the potential of HIVST and mHealth technology to overcome the barriers of *identification of candidacy* by making it easier for people to assess their candidacy for access to HIV testing and care services.

### Barriers to the Identification of Candidacy for HIV Testing and Linkage to Care

In this section, we focus on the barriers people face in recognizing themselves as *candidates* for HIV testing, treatment, or prevention. We highlight the complexities and multiple intersecting factors that shape people’s everyday experiences of accessing HIV services in their locations. 

#### Everybody Will Die One Day: Barriers to Individual Identification of Candidacy for HIV Testing

Our data show that it is difficult for participants, particularly men in both communities, to test for HIV. It seems some older women have transcended the challenges of HIV testing because of their engagement with sexual and reproductive health services. Men do not see themselves as candidates for HIV testing. They are more afraid of knowing their HIV status than HIV itself. This is partly because of the association of HIV with *assumed promiscuity* of men. Acquiring HIV is seen to be inevitable for men, and this is reflected in the below excerpt that everyone will die one day. The inevitability of HIV and the need to start HIV treatment one day are juxtaposed with the huge perceived social costs of knowing their HIV status (even if one identifies as a candidate), namely, discrimination and loss of loved ones, leading to delays in accessing HIV care services:

*With men there is no specific reason except that it’s a matter of reasoning...I would say they don’t have a concrete reason as to why they don’t want to. But they would say things like everybody has HIV or everybody will die one day, so am I, so why should I bother.*

IDI, M, 53, SP3

*They also don’t want to know their status; they try to avoid discrimination. They’re also afraid that they might lose their loved ones if they found out that they are HIV positive.*

IDI, Young Male, 26, CB

HIV-related stigma makes it more complex for some men to identify and assert their candidacy for HIV testing, treatment, or prevention, as they anticipate navigating the available HIV services until their candidacy is adjudicated by health care professionals. The pathway of accessing health care is an iterative process that requires an active response from users, whereas the barriers, people’s health needs, and perception of health services often create vulnerabilities [30,32]. For example, men (both young and old) are often seen as the major drivers of the HIV epidemic [34], and being seen at a local clinic, accessing HIV care, could reinforce suspicion, gossip, and stigmatization, even from local health care workers; therefore, men are reluctant to access HIV care services. As reported, some people (particularly men) are reluctant to test for HIV, as they will be judged to be HIV positive immediately when they are seen at their local clinic, even if just attending for testing. HIV-related stigma and unwillingness of men to utilize public clinic–based HIV testing and care services partly accounted for poor linkage to treatment in a TasP trial in KZN [7,11]. Our findings are supported by similar results from other sub-Saharan African studies on the impact of HIV-related stigma on people’s access to HIV testing, prevention, and care [12,13,32,35-39].

*Illness identity* is a crucial phase of individuals’ candidacy and multiple intersections that shape how it is navigated and adjudicated within the health care services. Previous studies have shown that people from disadvantaged settings are less likely to use preventive services and more likely to normalize *illnesses* because of their inability to see the positive side of health and their fear of being stigmatized or blamed by health care providers [30,33]. It was noted during the fieldwork that men are conscious of manliness being incompatible with illness; therefore, most of them living with HIV do not see themselves as candidates for HIV testing, and therefore do not know their status. Similar findings were reported about men across SA in a recent national HIV survey [4]. From the foregoing, perception of *HIV identity* and the blaming of men as drivers of the HIV epidemic make it more difficult for them to identify their candidacy for HIV testing and care, navigate the available HIV services, and appear at the clinic for their candidacy to be adjudicated by health care professionals. Our findings add to the knowledge and show that the fear of HIV-related stigma hinders people’s access to HIV services, despite the apparent normalization of HIV as an inevitable (and invisible) epidemic among men.

#### They Can’t Eat the Sweet While It’s on Paper: Association of HIV With Socially Undesirable Behaviors

Some participants, particularly young people (males and females), are afraid to test for HIV because of the way in which this could mark them out as having engaged in socially undesirable behaviors, such as unprotected sex, alcohol and drug abuse to health care providers, the community, and even peers. As reported by participants, the metaphorical *sweet*, which represents unprotected sex, is seen as ubiquitous in males, despite the association with HIV acquisition. Participants’ views are illustrated below:

From age 18 to age 28 [males and females], they are prone to be at risk of contracting HIV because you know they go to clubs and take alcohol and drugs...

IDI, Male, 30, SP4

Especially my friends...they say they can’t eat the ‘sweet while it’s on paper’ you see that thing, that’s the thing that can get them infected.

IDI, Young Male, 23, CA

Oh! the amount of unprotected sex that goes on here it astonishing and young kids just don’t understand; they don’t understand the implications of their actions...

IDI, Female, 34, SP2

This association of HIV acquisition with behaviors that are considered socially unacceptable and irresponsible, such as unprotected sex and sex under the influence of alcohol and drugs, makes it difficult for people to identify themselves as candidates for HIV testing at their local clinics. Specifically, for young people, it would be hard for them to identify themselves as candidates for HIV testing, even if they had not engaged in these socially undesirable behaviors, because of the widespread association of HIV with stigmatized behaviors in the community. As discussed above, identification of one’s candidacy for HIV testing is an important stage; however, participants continue to evaluate their candidacy on the basis of various factors, such as stigma. Research conducted elsewhere in rural SA further corroborates our findings that young people often delay seeking health care, particularly HIV testing, because of stigma [4,32,40].

### That Right Turn Is the Problem: Complexities of Navigation, Appearing at Services, and Adjudication

The layout of their local clinic, long queues, and inconveniences related to accessing services were described as significant barriers for participants in both communities. Most participants complained about the time required to access care. For men, clinic structure and operational hours are not suitable, given that most men in the locality are breadwinners, and they need to work to sustain their families:

If you go for [HIV] testing and you left home early in the morning, you will see that people [clinics] are full, and you may end up not doing it...

IDI, Older Male, 36, CB

However, inadvertent disclosure of HIV status and the ways in which people are treated by health care providers were also raised as barriers to care:

Here in community A, the clinic is split into two and when you go to the HIV section you have to take a right turn. That right turn is the problem because even someone that is passing by in a taxi can see that you are going there and will therefore immediately assume that you are HIV positive.

P3: FGD, Older Female, 43-67, CA

At the clinic people are complaining about the attitudes. After you have tested, other are being shouted at and you end up think that everyone is looking at you. They call your name “hey! Come this side,” people also fear that. And that a person would be seen by wrong people, I think this is the main challenge they face, it is difficult to go there.

IDI, Young Female, 25, CB

Our findings corroborate the findings of other studies that have described the structural barriers that hinder people’s (males and females) utilization of HIV services [41-43]. However, these structural barriers were compounded by anticipation that those who want to access HIV services are easily identified and stigmatized if they are seen going to a certain direction in the clinic. Finally, the attitudes of some health care workers (nurses) were frowned at. Almost all the participants in both communities complained about the inappropriate attitudes of some of the nurses at their local clinics. Our data validate and add nuances to growing evidence that HIV testing and treatment in public clinics are hampered by lack of privacy, long waiting times, segregation of services, and discrimination by health care workers [7,44-46]. Put together, the barriers hindering access to HIV testing and care services revealed the intersectionality of multiple factors affecting the recognition of one’s candidacy for HIV services, as well as health worker’s attitude as a key component of the *adjudication* process.

#### When You Tell Them to Go to the Clinic, They Say They are Scared: Community-General Anxieties to Accessing HIV Testing and Care

The fear of an *HIV identity* was common in both communities, and this is reflected in participants’ descriptions of *fear* of appearing at the clinic for HIV care services. Some participants are afraid of what people would say about them if they see them at the clinic accessing HIV care, whereas others fear finding out they are positive and the *unknown* life after. For example, a young woman said the following:

I don’t want to stress myself, because once I know my [HIV] status I will now start to think that I am going to die...

IDI, Young Female, 29, CA

Particularly, men fear the questions they will be asked about the person they date, her HIV status, and number of their sexual partners. Moreover, some men are uncomfortable seeking health care from their local clinics that are often dominated by female clients and health care providers, as they may not be treated as candidates for HIV testing and care services. For example, a young man maintains the following:

People don’t want to go to clinic because they [nurses] ask lot of questions because what I see is that they [mostly males] don’t like to be asked question, they are scared of being asked questions, maybe a person [nurse] will ask you how many people have you dated and what was their status?

IDI, Young Male, 21 CB

The fear of being stigmatized at the local clinics is also a challenge, as HIV care sections are often segregated. A young man shared his sentiment during the FGD:

When you get to the clinic to test for HIV, the rooms for HIV testing are excluded from other rooms.

**

These are small communities, and information circulates very fast; therefore, there are fears and anxieties of some people, particularly men and young people, about accessing HIV care services in their local communities. For some, knowing their HIV status seems like a death sentence; therefore, they refuse to test. As illustrated by the candidacy framework [30], the prediagnosis is an important phase of awareness or acceptance of one’s candidacy to access HIV testing and care services, which could be marred by barriers, such as fear of unknown consequences. Although we sought to understand how to improve people’s candidacy for HIV care, our findings correspond to the results of other studies conducted in similar environs and show that there is still a general anxiety when it comes to HIV testing and treatment, particularly among young people and men [7,38]. Similarly, an Eswatini study highlights that most men wait until their health deteriorates before they access HIV testing and care because of fear and stigma [12]. Given the local conditions and both internal and external stigma, the prediagnosis phase that was supposed to be the initial recognition of our participants’ candidacy to access HIV testing and care services was characterized by fear of an *HIV identity*. 

### Assessing Candidacy Through HIV Self-Testing and Mobile Phone–Enabled Linkage to Care

We now focus on the potential of HIVST and mHealth technology to identify candidates for HIV testing and facilitate linkage to care. We demonstrated HIVST and described the hypothetical functions of the proposed mHealth app to participants in the 2 communities. Our descriptions entail how they would conduct HIVST and be supported via the app, using mobile phone sensors (eg, camera) to interpret and display test results and then direct people to local clinics, to support virtual follow-up appointments and potentially provide access to antiretrovirals for both treatment and prevention (eg, Figure 1). A key finding discussed below is that an mHealth intervention can provide a way for people to test their candidacy for HIV service, which may eventually help to overcome the first barrier in the candidacy cascade. 

#### Potential of HIV Self-Testing to Identify Candidates for HIV Testing

Generally, participants valued *the potential privacy and convenience* associated with HIVST. Given that HIV-related stigma is widespread in both communities studied, HIVST is generally acceptable, particularly among men and young people, as it gives them control of HIV testing, privacy, and convenience, thereby attracting candidates who were previously deterred by clinic-based testing. Participants are generally enthusiastic about HIVST, as reflected in the following quote from a 23-year-old young man:

I think it [HIVST] gives you that braveness that you test yourself.

A young woman also shared her sentiments about HIVST:

It [HIVST] will help me because, going to the clinic takes time, hence it will save time and money for transport...

IDI, Young Female, 23, CB

Our data show participants’ high willingness to perform HIVST, and this may be a strategy to identify candidates and improve diagnosis by placing HIV testing and disclosure within individuals’ control. Similarly, HIVST appears highly acceptable in other settings because of its convenience and privacy, despite concerns about counseling and linkage to care [16,20,47].

However, there is a general concern about social harms, such as suicide, depression, and alcoholism, which could come with HIVST. These concerns about HIVST are not peculiar to our study, as they have been documented in other studies [48,49]. A few participants raised concerns that people may commit suicide after testing HIV positive without health care provider support, but this was deemed less of an issue by providers, given the high level of community awareness about HIV management. Abusing alcohol was seen more as a way of coping with their *HIV+ identity*, as this is widely stigmatized in both communities, than a consequence of HIVST:

I don’t think suicide is a problem now, I think alcoholism. It [HIV+ status] drives them to that now…that’s how they try to deal with it. I don’t think people will kill themselves I know a lot of people that are sick [HIV+], and they do get depressed.

IDI, Female, 28, SP1

Thus, HIVST may allow people (particularly men and young people) to explore their candidacy for HIV testing in private and overcome fears of being assumed to be positive even if one has a negative test in clinic, but it does not erase the fear of the consequences of an *HIV identity* and other external factors or stigma [50]. Despite people’s concerns and anxieties around new HIVST technology, several studies and systematic reviews have shown high uptake of HIVST, as well as its potential to attract first-time testers, particularly hard-to-reach populations [16,20,47,51].

#### Potential of Mobile Health Intervention to Overcome the Barriers of Individual Candidacy for HIV Care Services

This theme explores the potential of a mobile phone–connected Web-based clinical care pathway to link people to HIV prevention and care. Mobile phone–enabled linkage to care was broadly acceptable to potential users (including providers) because of the ability to test their candidacy for HIV care in private and at their own convenience. Participants, particularly men and young people, believe that linking to HIV care services through their phones provides opportunities to save time and reduce experienced stigma. The privacy and convenience the proposed app may provide is reflected in the following excerpts:

I think it is a good idea that there is technology like this. And I think it will work mostly for young people, because youth are the people that mostly get infected with HIV...Yes, I think they will be motivated, because it entails the privacy. Hence, no one will see other people’s results since people will be testing alone. Then this APP will link with the clinic, then the clinic will continue to help you...

IDI, Young Female, 30, CA

Because it will be easy by that time to know yourself and not be scared... because the time you link with the clinic you will be able to make an appointment to collect your pills, and if you come to the clinic because you have made an appointment you will just walk in and take your pills and go maybe that can help and prevent the loss of self-confidence I think it will work a lot and I know young people they like to get into things like that.

IDI, Young Male, 26, CB

To participants, the proposed app, coupled with HIVST, could be a game changer by providing a way for an individual to identify and test their *candidacy* for HIV services. The particular appeal of the intervention was that it could overcome the experience of stigma and structural barriers of clinic-based testing by providing privacy and convenience. However, it does not completely erase individuals’ fear of an HIV identity (self-stigmatization), as well as the barriers of navigating and appearing at HIV services for further care. The barriers that make the services unattractive at fixed clinic level will need to be addressed if potential demand is to turn into actual demand. Moreover, the development, implementation, and evaluation of the app will provide data about its receptivity and usage, as technology *likeness* does not mean that an individual automatically identifies as a candidate for its use.

Despite the declared willingness of participants to use the technology, a few concerns were raised about digital literacy and data consumption. Some participants raised concerns that some people would be left out, as they cannot afford smartphones, whereas others (particularly old people) with a smartphone cannot navigate it effectively, which could impede the assertion of their candidacy for the service. The concern around literacy and linkage is reflected in the following excerpt from a male SP:

Yeah, my concern is that if people are not well informed about the service that is linking the client after the self-test, then there might be a problem...

The concern on data is reflected in the following excerpts from an older male FGD participant:

Where can we find the money to access this app? The problem will be airtime.

These echo concerns around how technology and the digital divide can increase rather than reduce health inequalities [20].

Most participants were enthusiastic and willing to use the proposed app that has the potential to provide access to real-time HIV care, interpreting test result, counseling, relevant health information, and further linkage. Our results correspond to the findings of a recent study conducted with men who have sex with men (MSM) in China, using WeChat (app) to identify and assert their candidacy for HIV testing, prevention, and care. That study found that MSM who performed HIVST in private (eg, at home) and tested positive sought support via the app in interpreting their HIV results and linkage to treatment [20]. Other studies have also shown that software apps have the potential to reach and encourage hard-to-reach populations to assert their candidacy for a particular service [52,53]. Owing to the self-sufficient nature of HIVST in which individuals can test and interpret their HIV results in a private location without the support of a trained counselor, the proposed app will allow people to test whether they are candidates for HIV care, and it may subsequently facilitate HIV testing and linkage to treatment if users see themselves as candidates for the service. 

#### Partnership for Change: Community Cocreation of Mobile Health Intervention to Improve Access to HIV Care Services

To cocreate a user-friendly mHealth technology that could be used at home and in clinic settings to overcome some of the barriers identified above, end users and health care providers were asked about additional features they would like to see in the proposed app and whether they are happy to receive health-related messages on their mobile phones. Generally, participants were enthusiastic to receive health messages on their personal mobile phones and happy to describe features they believed would support them assess their candidacy for testing and care. In keeping with other studies, they found the concept empowering, convenient, and with the potential to reduce the experience of stigma [20,54,55]. Key features were therefore to reduce the time spent at the clinic and support privacy and confidentiality, as reflected in these excerpts:

It’s good because sometimes I need to go to the clinic, yet I don’t go because I am scared of all the people. If I get a message on my phone notifying me of my parcel’s [HIV medication] readiness, I can go to the clinic knowing that I will be in and out quickly.

P2: FGD, Older Female, 36-48, CB

I think the counselling one must be there because counselling make person to be positive about that thing…That’s why the App must be locked, you will go there and put your personal details so the other person can’t access your information because that will mean your information is open to everyone if it will be just the password only, there must also be other things like questions that will be asked like a confirm questions. It can ask for your mother’s name or your relative...

P1: FGD, Young Male, 18-28, CA

Participants in our study tried to limit physical contact with health facilities; therefore, they were interested in the alternative method that is private and confidential and that would help in asserting their candidacy by easing their access to HIV testing and care services. In parallel though, participants wanted counseling and support for some people to be able to cope if they test positive for HIV. This acknowledgment of the need for *counseling and support* via the app is consistent with findings of other studies [20,54]*.* Our findings show that participants are important partners in cocreating a sustainable intervention that will improve the health outcomes of the community.

### Study Limitations and Strengths

Owing to the specific study sites, sampling, and sample size, generalizability of the qualitative results to other settings in uMkhanyakude district or outside KZN province may be limited. Some findings presented in this paper were based on a *hypothetical mHealth intervention* described to participants; therefore, assessment of actual receptivity and usage of the app for linkage to care and its impact on HIVST behaviors needs to be conducted after it has been developed, as this may differ from how people imagined it to be. Owing to the ever-changing nature of mobile technology and user expectations in app-based communications, the specific recommendations reported in this paper about the proposed app for HIVST promotion and self-administration may have time-limited relevance. Nevertheless, the preformative and main research data gathered from 2 different communities highlight the strength of the study, as it afforded us the opportunity to draw on a range of experiences across 2 sites. The differences and similarities in the experiences of participants across the sites were invaluable and add nuance to our understanding of the current situation, as well as the potential to codevelop (with the community including health care providers) a suitable mHealth intervention to improve people’s health outcomes in a resource-constrained setting.

## Discussion

Overall, the study shows that mHealth intervention developed with potential end users, including health care providers, may allow people to explore and test their candidacy for HIV testing and treatment or prevention. However, it cannot overcome the fear of an *HIV identity* (self-stigmatization) and other factors (eg, external stigma) that permeate people’s everyday lives, making it difficult for them to assert their candidacy without improving the quality of HIV care available. Features of HIVST and mobile phone–connected Web-based clinical pathways that have the potential to overcome some of the social costs of HIV testing and treatment are their privacy and convenience, as well as allowing the user to be in control of the process. However, there are residual anxieties around digital literacy and the potential to increase health inequalities. mHealth interventions’ greatest potential will be to support decentralizing HIV testing, care, and prevention services from clinics to key places that are male- and youth-friendly in the community or workplace where men or young people can be reached. To ensure its effectiveness in reducing HIV incidence and mortality, work still needs to be done to tackle the social norms that continue to fuel stigma and the fear of an *HIV identity*.

## Abbreviations

ART: antiretroviral therapy
CA: first community
CB: second community
FGD: focus group discussion
HIVST: HIV self-testing
IDI: in-depth interview
KZN: KwaZulu-Natal
mHealth: mobile health
MSM: men who have sex with men
PrEP: Pre-Exposure Prophylaxis
SA: South Africa
SP: service provider
TasP: treatment as prevention
UTT: Universal HIV test and treat
